# Cerebral and Systemic Stress Parameters in Correlation with Jugulo-Arterial CO_2_ Gap as a Marker of Cerebral Perfusion during Carotid Endarterectomy

**DOI:** 10.3390/jcm10235479

**Published:** 2021-11-23

**Authors:** Zoltán Kovács-Ábrahám, Timea Aczél, Gábor Jancsó, Zoltán Horváth-Szalai, Lajos Nagy, Ildikó Tóth, Bálint Nagy, Tihamér Molnár, Péter Szabó

**Affiliations:** 1 Department of Anesthesiology and Intensive Care, Medical School, University of Pécs, H-7624 Pécs, Hungary; kovacsabrahamzoltan@gmail.com (Z.K.-Á.); itoth2777@yahoo.com (I.T.); balintjanosnagy@yahoo.com (B.N.); tihamermolnar@yahoo.com (T.M.); 2 Department of Pharmacology and Pharmacotherapy, Medical School, University of Pécs, H-7624 Pécs, Hungary; aczel.timea@pte.hu; 3 Molecular Pharmacology Research Group & Centre for Neuroscience, János Szentágothai Research Centre, University of Pécs, H-7624 Pécs, Hungary; 4 Department of Vascular Surgery, Medical School, University of Pécs, H-7624 Pécs, Hungary; jancso.gabor@pte.hu; 5 Department of Laboratory Medicine, Medical School, University of Pécs, H-7624 Pécs, Hungary; horvath-szalai.zoltan@pte.hu; 6 Department of Applied Chemistry, Institute of Chemistry, Faculty of Science and Technology, University of Debrecen, H-4032 Debrecen, Hungary; nagy.lajos@science.unideb.hu

**Keywords:** carotid endarterectomy, carbon dioxide gap, cerebrovascular circulation, cortisol, L-arginine, S100B, frailty, cervical plexus block

## Abstract

Intraoperative stress is common to patients undergoing carotid endarterectomy (CEA); thus, impaired oxygen and metabolic balance may appear. In this study, we aimed to identify new markers of intraoperative cerebral ischemia, with predictive value on postoperative complications during CEA, performed in regional anesthesia. A total of 54 patients with significant carotid stenosis were recruited and submitted to CEA. Jugular and arterial blood samples were taken four times during operation, to measure the jugulo-arterial carbon dioxide partial pressure difference (P(j-a)CO_2_), and cortisol, S100B, L-arginine, and lactate levels. A positive correlation was found between preoperative cortisol levels and all S100B concentrations. In addition, they are positively correlated with P(j-a)CO_2_ values. Conversely, postoperative cortisol inversely correlates with P(j-a)CO_2_ and postoperative S100B values. A negative correlation was observed between maximum systolic and pulse pressures and P(j-a)CO_2_ after carotid clamp and before the release of clamp. Our data suggest that preoperative cortisol, S100B, L-arginine reflect patients’ frailty, while these parameters postoperatively are influenced by intraoperative stress and injury. As a novelty, P(j-a)CO_2_ might be an emerging indicator of cerebral blood flow during CEA.

## 1. Introduction

Carotid endarterectomy (CEA) is a widely accepted method for the treatment of severe carotid stenosis and stroke prevention. During CEA, carotid cross-clamping is performed [1]. CEA is often associated with increased intraoperative stress [2,3]. Patients with vascular disease are especially sensitive to distress. Frailty and postoperative complications commonly occur among them [4]. During cross-clamping, the risk of cerebral hypoperfusion is present [5]. Symptomatic ischemia is most certainly detected on vigilant patients by simply evaluating the verbal and contralateral motor functions [6,7,8]. If a sudden neurological symptom appears, shunt insertion—as a temporary bypass—is applied to avoid ischaemic injury [9]. To adequately monitor cerebral function, CEA is often performed in regional anesthesia, most frequently by cervical plexus block [10]. Besides the general advantages [11], operating on awake patients offers hemodynamic benefits, e.g., fewer hypotensive episodes, and thus less vasopressor usage, and consequently better cerebral perfusion [12,13,14,15].

Cortisol’s plasma concentration rises rapidly due to intraoperative stress [16,17]. We described cortisol’s dynamics in the perioperative period of CEA in our previous investigation [18]. The rate of cellular anaerobic metabolism correlates with serum lactate levels [19]. S100B is a calcium-binding astroglial protein considered to be an indicator of blood–brain barrier injury and disruption [20,21]. S100B concentration rises after previous cerebral vascular events [22] and is also related to uneventful periprocedural brain injuries [23,24,25,26]. In addition, it might have an important role in the regulation of vascular tone after brain ischemia [27].

Arterial and venous blood gas sample measurements provide additional information on impaired oxygen and metabolic balance [28,29]. Venoarterial carbon dioxide differences (P(v-a)CO_2_) are a marker of tissue hypoperfusion and can be the target of fluid resuscitation in circulatory failure, hypovolemic and septic shock [30,31,32]. It is also used for recognizing cerebral ischemia [33,34,35,36]. We hypothesized that blood gas-derived parameters from the jugular vein and arterial samples provide information about anaerobic metabolism and ischemic stress of the brain throughout the procedure. L-arginine is an important donor of nitric oxide (NO), which causes vasodilation, while asymmetric dimethylarginine (ADMA) and symmetric dimethylarginine (SDMA) antagonize its effect. Nitric-oxide pathway proteins’ role in the brain’s circulation and their association with neuroprotection and outcome is comprehensively examined [37,38,39,40,41,42]. In our previous study, we had found that low L-arginine concentration is related to a higher risk of shunt insertion during CEA [43].

We hypothesized that there might be minor ischemic events, which are difficult to identify, but which may still increase the distress of patients. They potentially increase the risk of other postoperative complications. Our main purpose was to describe the relation between indicators of brain perfusion (P(j-a)CO_2_ and lactate), potential cerebral injury (S100B), and perioperative stress (cortisol).

## 2. Materials and Methods

### 2.1. Patient Recruitment

This prospective, observational clinical study was approved by the Clinical Centers Regional and Institutional Research Ethics Committee, N°4820. Data were extracted from a formerly recruited prospective database and analyzed retrospectively [43]. Informed consent was obtained from each patient.

A total of 54 consecutive patients with significant carotid stenosis (between 70% and 95%) were recruited into this study at the Department of Vascular Surgery at the University of Pécs. The diagnosis of significant carotid stenosis was based on both carotid duplex ultrasound and CT angiography. Narrowing of the lumen between 70% and 99% was considered as significant carotid stenosis.

### 2.2. Anesthesia and Neuromonitoring

All operations were performed in regional anesthesia. Deep and superficial cervical plexus blocks were performed in each patient, using 20 mL bupivacaine 0.5% and 10 mL lidocaine 1%. Additional local anesthetic, if necessary, was given by the surgeon. The patient’s awareness, verbal responsiveness, and the contralateral hand’s motoric function were evaluated. Any sudden decline in Glasgow coma score after clamp of carotid artery indicated shunt insertion.

### 2.3. Blood Sampling Protocol and Laboratory Measurements

An arterial line was inserted by the anesthetist in the operating theatre. The internal jugular vein was directly cannulated by the operating surgeon, the tip of the catheter was positioned in the jugular bulb. Jugular and arterial blood gas samples were taken four times during operation: before carotid clamp (T1), 3 min after clamp (T2), 1 min before (T3), and 1 min after releasing the clamp (T4). Arterial and jugular venous blood gas analysis and lactate measurements were made at each time point by Radiometer Abl 800 flex (Copenhagen, Denmark).

Arterial samples for the measurement of L-arginine, S100B, and cortisol were taken in lithium heparin tubes (1.) before the beginning of the operation, in the operation room, (2.) before, and (3.) after the release of the carotid clamp, and (4.) 2 h after the operation.

After centrifugation at 3000× *g* for 10 min, plasma samples were frozen within 60 min and stored at −80 °C until analysis, as previously described [18,40,43]. Briefly, levels of S100B were examined by automated electrochemiluminescent immunoassay (Liaison Sangtec 100 system; DiaSorin, Bromma, Sweden). The amino acid content of the blood serum samples was retrieved by the solid-phase extraction (SPE) method and was quantified by high-performance liquid chromatography after derivatization. Arginine and homoarginine were detected at ʎ = 337 nm, ʎ = 520 nm. Analysis of plasma cortisol levels was performed by a commercially available solid phase, competitive chemiluminescent immunoassay (Immulite 2000 Siemens, Healthcare Diagnostics, Erlangen, Germany). All measurements were performed in duplicate.

Jugulo-arterial carbon dioxide tension difference (P(j-a)CO_2_) as a potential marker of anaerobic metabolism and perfusion was calculated using the following formula:

P(j-a)CO_2_ = PjCO_2_ − PaCO_2_.

In addition, we have calculated the change of jugulo-arterial lactate difference between T4 and T1 as a parameter for “lactate production”:

ΔLact(j-a)T4-T1 = (LactjugT4-LactartT4) − (LactjugT1-LactartT1).

All parameters were measured at all specific time points during the perioperative period. Blood gas-derived variables and lactate were measured only during the operation.

All patients’ maximum and minimum blood pressures were recorded throughout the procedure. Maximum and minimum systolic-diastolic differences, as pulse pressures, were counted.

### 2.4. Statistical Analysis

Data were evaluated using SPSS (version 22.0; IBM, Armonk, NY, USA). Categorical data were summarized using means of absolute and relative frequencies (counts and percentages). Quantitative data are presented as mean and 95% confidence interval (CI), as well as mean ± SD. The Kolmogorov–Smirnov test was applied to check for normality. The chi-square test for categorical data and student t-test for continuous data were used for the analysis of demographic and clinical factors. Correlation analysis was performed calculating Spearman’s correlation coefficient (r). A *p*-value < 0.05 was considered statistically significant.

## 3. Results

### 3.1. Clinical Characteristics

Patients’ demographic and clinical characteristics are shown in Table 1.

Among 54 patients, 5 were shunted during operation. However, there were no between-group differences in age, body mass index (BMI), or any other parameters measured.

### 3.2. Temporal Characterization of Jugulo-Arterial Carbon Dioxide Tension Difference (P(j-a)CO_2_)

**Figure 1:**
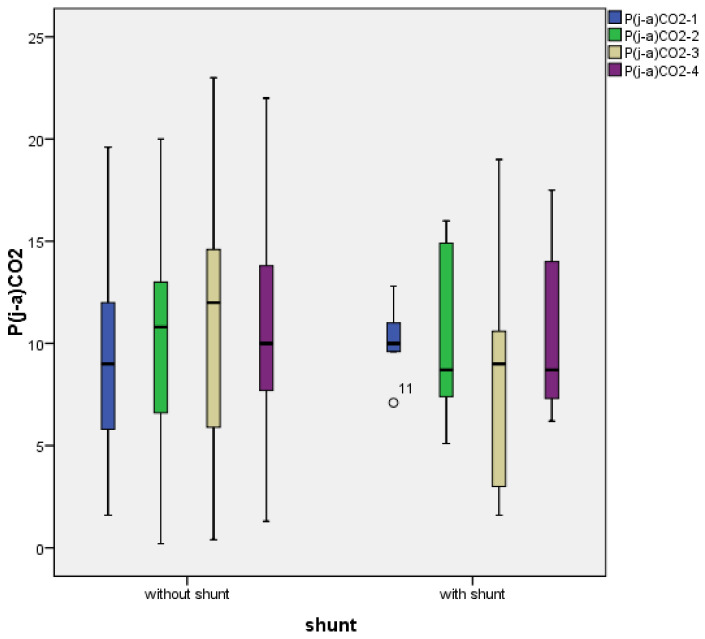
Jugulo-arterial carbon dioxide gap (P(j-a)CO_2_) dynamics through the operation, in four different time points: T1 (blue), T2 (green), T3 (yellow), and T4 (purple). P(j-a)CO_2_ (mmHg) is expressed as a box-plot diagram.

The temporal profile of P(j-a)CO_2_ through the operation is demonstrated in Figure 1. In patients without shunt the value of P(j-a)CO_2_ increases until the end of the carotid clamp and decreases after the release of clamp. The opposite is seen in shunted patients, where P(j-a)CO_2_ decreases after carotid clamp and shunt insertion. After reperfusion (T3), the value of P(j-a)CO_2_ was lower than at T1.

### 3.3. Jugulo-Arterial Carbon Dioxide Tension Difference (P(j-a)CO_2_) and Cortisol’s Relation

Cortisol measured at the beginning of the operation showed a positive correlation with P(j-a)CO_2_ values counted after carotid clamp, before and after release of clamp (P(j-a)CO_2_-2: r = 0.334, *p* = 0.043; P(j-a)CO_2_-3: r = 0.406, *p* = 0.013; P(j-a)CO_2_-4: r = 0.433, *p* = 0.007).

In contrast, cortisol measured 2 h after CEA inversely correlated with P(j-a)CO_2_ counted before and after carotid clamp and before release of clamp (P(j-a)CO_2_-1: r = −0.278, *p* = 0.048; P(j-a)CO_2_-2: r = −0.276, *p* = 0.05; P(j-a)CO_2_-3: r = −0.421, *p* = 0.002). The correlation was not significant after the release of clamp (P(j-a)CO_2_-4).

**Figure 2:**
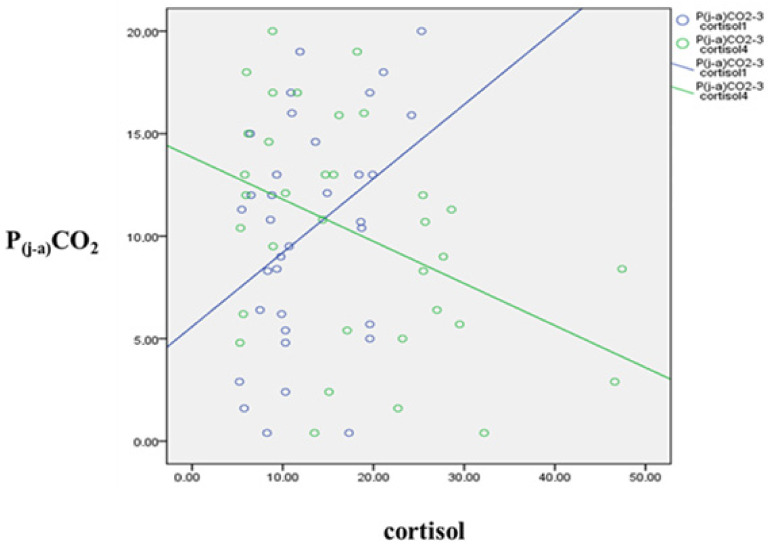
Scatter plot showing a correlation between P(j-a)CO_2_ (mm Hg) and cortisol (nmol/L) levels before and after the operation. The circles represent the plotted values for each of the variables while the lines represent the best fit for the correlation between them. Blue line represents the correlation of P(j-a)CO_2_ before release of clamp and preoperative cortisol, Green line represents the correlation of P(j-a)CO_2_ before release of clamp and cortisol 2 h after the operation.

Examples for the reversing tendency of cortisol and P(j-a)CO_2_ correlations (cortisol-1—P(j-a)CO_2_-3, cortisol-4—P(j-a)CO_2_-3) are presented in Figure 2.

### 3.4. S100B’s Relation to Jugulo-Arterial Carbon Dioxide Gap (P(j-a)CO_2_) and Cortisol

Baseline S100B directly correlated with P(j-a)CO_2_ at all time points (P(j-a)CO_2_-1: r = 0.394, *p* = 0.003; P(j-a)CO_2_-2: r = 0.270, *p* = 0.048; P(j-a)CO_2_-3: r = 0.306, *p* = 0.024; P(j-a)CO_2_-4: r = 0.277, *p* = 0.043).

Moreover, baseline S100B directly correlated with baseline cortisol (r = 0.402, *p* = 0.014) and inversely with cortisol after reperfusion (r = −0.456, *p* = 0.005), at the end of the operation.

**Figure 3:**
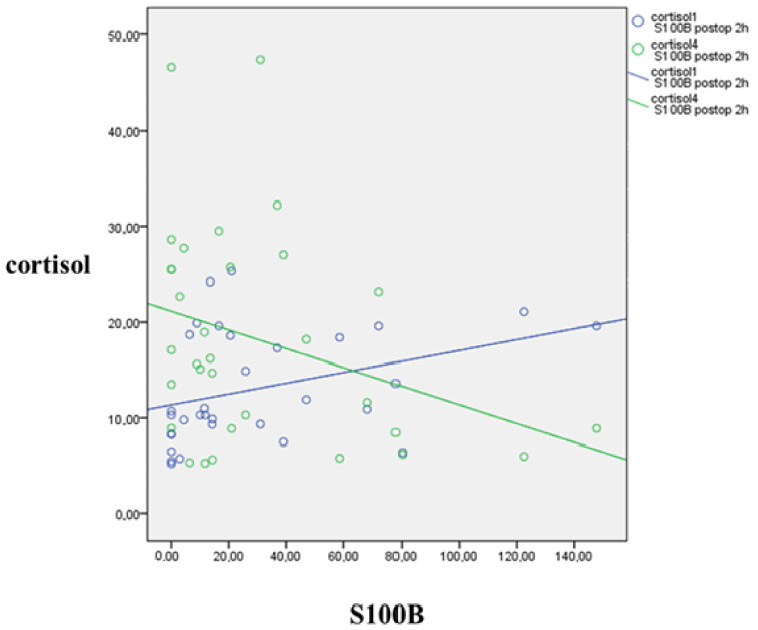
Correlation between concentrations of postoperative S100B (nmol/L) and cortisol (nmol/L) before and after the operation. The circles represent the plotted values for each of the variables. Blue line represents the correlation of preoperative cortisol and S100B 2 h after the operation. Green line represents the correlation of cortisol 2 h after the operation and S100B 2 h after the operation.

S100B measured 2 h after the operation showed a positive correlation with baseline cortisol (r = 0.471, *p* = 0.006) and negative correlation with cortisol measured 2 h postoperatively (r = −0.295, *p* = 0.040), as shown in Figure 3.

### 3.5. Correlations between Lactate, S100B, Cortisol and L-Arginine

Interestingly, there were no statistical correlations between any of the P(j-a)CO_2_-values and lactate concentrations.

Change in jugulo-arterial lactate difference between T4 and T1 directly correlated with S100B values measured before (r = 0.301, *p* = 0.027) and after the release of clamp (r = 0.278, *p* = 0.042).

The baseline L-arginine concentration inversely correlated with the baseline arterial lactate concentration (r = −0.480, *p* = 0.001), and with the postoperative 2 h S100B concentration (r = −0.367, *p* = 0.017), and consequently with all subsequent lactate concentrations. By the end of the operation, cortisol and L-arginine had an inverse correlation (r = −0.560, *p* = 0.037).

### 3.6. Correlation of Blood Pressure and P(j-a)CO_2_

**Figure 4:**
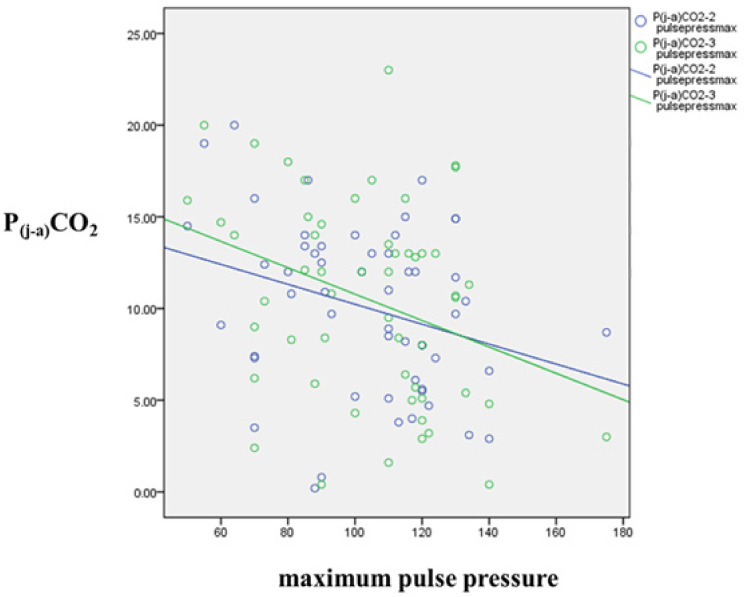
Correlation between maximum pulse pressure and P(j-a)CO_2_ (mm Hg) during the carotid clamp. The circles represent the plotted values for each of the variables while the lines represent the best fit for the correlation between them. Blue line represents correlation of maximum pulse pressure and P(j-a)CO_2_ after carotid clamp. Green line represents the correlation of maximum pulse pressure and P(j-a)CO_2_ before release of clamp.

A negative correlation was observed between maximum systolic (max. syst.) and pulse pressures and P(j-a)CO_2_ after carotid clamp and before the release of clamp—while the carotid artery was clamped, through the period of hypoperfusion (max. syst. pressure and P(j-a)CO_2_-2: r = −0.296, *p* = 0.030; max. syst. pressure and P(j-a)CO_2_-3: −0.314, *p* = 0.021; max. pulse pressure and P(j-a)CO_2_-2: r = −0.305, *p* = 0.025; max. pulse pressure and P(j-a)CO_2_-3: r = −0.332, *p* = 0.014). These associations are displayed in Figure 4.

A positive correlation was seen between postoperative 2 h cortisol and maximum pulse pressure (r = 0.363, *p* = 0.009).

## 4. Discussion

To check the association between the theoretically harmful perioperative stress [5,15,44] and brain metabolism, we decided to analyze the correlation of multiple parameters potentially related to stress, frailty and cerebral injury.

It is important to note that in each case of severe cerebral hypoperfusion after the carotid artery clamp, an intravascular shunt was inserted and the brief ischemic period was immediately terminated. No prolonged cerebral ischemia occurred. Therefore, only low-grade lactate alterations were seen. In contrast, patients with a poor vascular state tend to have impaired tissue circulation, thus higher systemic lactate concentrations. The deficient general circulation might be associated with the brain’s increased sensitivity for hypoperfusion [45]. We were not able to measure the exact volume of blood supplying the brain. Only the directions of alterations are known, as it decreases after the carotid clamp and increases after the release of clamp.

Jugulo-arterial carbon dioxide difference or “carbon dioxide gap” (P(j-a)CO_2_) is a promising parameter for identifying discrepancies of cerebral circulation [33,34,35]. It is mostly determined by jugular venous carbon dioxide tension, which is influenced by the production of CO_2_ in brain cells and elimination by cerebral blood flow. More CO_2_ is produced during aerobic circumstances than in anaerobic conditions [46]. Thus, theoretically, jugular carbon dioxide tension should increase; however, there was no correlation between any of the P(j-a)CO_2_ and lactate values. The possibility of major cellular anaerobic circumstances can be excluded in awake patients without acute neurological symptoms [47]. Conversely, in case of the perfusion’s decline, the lower amount of blood becomes more saturated with CO_2_. Since intraoperative P(j-a)CO_2_ tends to be lower in shunted patients (in whom the cerebral circulation is unharmed for the period of the carotid clamp), perfusion seems to have a greater impact on jugular pCO_2_ (Figure 1).

From all of the blood gas-derived parameters, P(j-a)CO_2,_ showed some notable statistical associations. In non-shunted patients, P(j-a)CO_2_ increases during carotid clamp and decreases after the release of the clamp, although it remains higher than the baseline (T1) value after reperfusion. After the release of the clamp, the greatly improved blood flow washes out the CO_2_ from the brain and immediately dilutes it. Thus, from T3 to T4 P(j-a)CO_2_ decreases, but still exceeds T1 values. At T4, due to the significantly improved cerebral blood flow, the high value of P(j-a)CO_2_ represents a considerably larger quantity of CO_2_ than at T1. Consequently, during the carotid clamp, more CO_2_ has to remain in the tissues since the reduced circulation is not able to transfer it. Thus, it seems that hypoperfusion is present through the clamp, even without any apparent symptoms. This phenomenon could not be observed in shunted patients. In this case, after shunt insertion the P(j-a)CO_2_ decreases. At reperfusion, P(j-a)CO_2_ becomes even lower than at the beginning of the operation, demonstrating the integrity of cerebral circulation throughout the carotid clamp. Briefly, the correlation is negative between P(j-a)CO_2_ and the quality of cerebral perfusion. The higher values of P(j-a)CO_2_ are referring to reduced perfusion, even without noticeable symptoms.

Cortisol concentration might be not only the indicator of patients’ actual stress, but may represent a kind of general frailty [48]. The increased baseline cortisol correlation with increased P(j-a)CO_2_ indicates worse brain perfusion. Surprisingly, higher postoperative cortisol is associated with lower P(j-a)CO_2_ and consequently better perfusion. Theoretically, this reversed correlation could be explained by the protective effect of higher blood pressures related to the increased stress [49]. The positive correlation between postoperative cortisol and both maximum pulse pressure and maximum systolic pressure supports the hypothesis of increased stress’ beneficial effect. This is in line with literature data characterizing cortisol’s effect on blood pressure [12,13,17]. There is a negative correlation between maximum systolic- and pulse pressure and P(j-a)CO_2_ when the carotid artery is clamped, during the period of impaired cerebral blood flow. Postoperative cortisol, a possible indicator of overall procedural stress, is linked to increased blood pressure. Furthermore, higher blood pressure results in better perfusion (concluded from P(j-a)CO_2_) through the entire period of the carotid artery clamp. It has been a long-time recommendation to keep blood pressure elevated during CEA [14,15,50]. Our observations support these suggestions.

The brain’s oxygen demand is feared to be higher in awake patients when regional anesthesia is chosen [50]. Moreover, stress is often anticipated to increase the risk of cerebral ischemia by worsening cerebral vasoconstriction and the risk of neurological injury is expected to be grown [51]. Our results do not provide evidence for these preconceptions. We could not find any statistical relationship between stress and arterio-jugular blood oxygen content difference (oxygen extraction), which could prove the theoretically higher oxygen consumption. Conversely, the increased stress linked with higher blood pressure seems to have a protective role in terms of cerebral ischemia.

Elevated preoperative S100B, considered to be the sign of former cerebral ischemic events, is associated with higher P(j-a)CO_2_ (impaired perfusion), increased baseline cortisol, and decreased cortisol concentration by the end of the operation. The latter correlation could be explained either by the depressed ability to respond to stress due to frailty or by the better adaptation to stress after a previous injury. S100B may play a role in vascular adaptation after both severe and uneventful injuries [21,23,27].

A similar reversing correlation was seen between postoperative S100B and cortisol, measured baseline and postoperatively. The change of jugulo-arterial lactate difference between the end and beginning of the operation, a possible indicator of “lactate production” is directly related to S100B by the end of the operation. Hence, the grade of hypoperfusion during clamp appears to have an impact on postoperative S100B values. Frail patients have higher baseline cortisol concentrations and are more sensitive to cerebral ischemia [52]. Higher postoperative S100B is correlated with lower cortisol, measured at the same time point, which probably could be explained by the lower blood pressure associated with lower stress and with the consequent silent ischemia.

Inverse correlation between L-arginine and cortisol by the end of procedure shows the connection of circulation and stress. The protective function of L-arginine is already known [38,39,40,43,53]. Better cerebral circulation seems to have a beneficial effect on operation-related distress. In accordance with our previous results [43], L-arginine has a negative correlation with lactate- and S100B concentrations.

In shunted patients, concentrations of baseline arterial lactate, and cortisol by the end of the operation and 2 h postoperatively are significantly higher, L-arginine is lower. In non-shunted patients, the baseline cortisol concentration was positively related to the lactate values at all timepoints.

The most important measured baseline variables like arterial lactate, cortisol, and S100B tend to demonstrate some kind of vulnerability, a former injury or impaired vascular state. The same derivates measured later, after the operation, can provide information about silent periprocedural injuries.

In summary, P(j-a)CO_2_ inversely correlates with the quality of the brain’s blood flow, therefore it can be an adequate parameter to describe cerebral perfusion. The carotid artery’s clamp is potentially associated with a silent hypoperfusion. Our data also suggest that blood pressure plays a role in maintaining appropriate brain perfusion during CEA. In addition, higher cortisol and S100B might indicate a protective effect against distressing factors. The current study highlights that higher baseline arterial lactate, S100B and cortisol, and low L-arginine concentrations possibly predict patient’s increased vulnerability to stressor agents. In this study, we emphasize the relationship of jugulo-arterial carbon dioxide tension difference (P(j-a)CO_2_) to cerebral perfusion, intraoperative stress and ischaemia during CEA.

## Figures and Tables

**Table 1:** Demographics and clinical factors.

	Total Population *n* = 54	Without Shunt *n* = 49	Shunted Patients *n* = 5	*p*-Value
age	65.59 ± 8.0	65.45 ± 8.31	67 ± 4.18	0.504
male (%)	43 (79.6)	38 (77.55)	5 (100)	0.206
BMI	27.33 ± 4.57	27.12 ± 4.59	29.24 ± 4.37	0.353
right sided operation (%)	23 (42.59)	22 (44.89)	1 (20)	0.658
operated stenosis (%)	84.72 ± 6.33	84.9 ± 6.25	83.0 ± 7.58	0.614
contralateral stenosis (%)	44.89 ± 25.74	45,35 ± 25.71	40 ± 29.44	0.746
clamp time (minutes)	22.11 ± 6.36	22.36 ± 6.17	19.66 ± 8.4	0.520
creatinine (µmol/L)	81.37 ± 22.25	80.53 ± 22.13	89.6 ± 24.2	0.460
hemoglobin before surgery (g/dL)	14.19 ± 1.09	14.14 ± 1.13	14.76 ± 0.54	0.062
hemoglobin after surgery (g/dL)	12.98 ± 1.02	12.95 ± 1.02	13.24 ± 1.12	0.607
Previous stroke (%)	20 (37)	18 (36.7)	2 (40)	0.885
Previous TIA (%)	10 (18.5)	9 (18.4)	1 (20)	0.929

BMI: body mass index. P: *p*-value for statistical significance. Data are presented as mean ± SD or absolute number (percentage).

## Data Availability

ClinicalTrials.gov Identifier: NCT03957018. The data that support the findings of this study are available from the corresponding author upon reasonable request.
